# A relational investigation of Israeli gay fathers’ experiences of surrogacy, early parenthood, and mental health in the context of the COVID-19 pandemic

**DOI:** 10.1371/journal.pone.0282330

**Published:** 2023-02-24

**Authors:** Hannah Kate Kelly, Shulamit Geller, Viren Swami, Geva Shenkman, Sigal Levy, Damien Ridge

**Affiliations:** 1 School of Social Sciences, University of Westminster, London, United Kingdom; 2 Statistics Education Unit, The Academic College of Tel Aviv Yaffo, Tel Aviv-Yafo, Israel; 3 School of Psychology and Sport Science, Anglia Ruskin University, Cambridge, United Kingdom; 4 Centre for Psychological Medicine, Perdana University, Kuala Lumpur, Malaysia; 5 School of Psychology, Reichman University (Interdisciplinary Center, IDC), Herzliya, Israel; Walden University, UNITED STATES

## Abstract

Perinatal distress affects approximately 10% of fathers, but little is known about how gay fathers experience the challenges surrounding childbirth and early parenting of a child. This study explored gay fathers’ experiences of having a baby via transnational surrogacy, raising that baby as a gay parent, and the context of the COVID-19 pandemic. In-depth qualitative interviews were conducted with 15 Israeli men to understand their experiences of surrogacy and early parenthood, focusing on the impact on their mental health and the relational factors involved. Secondary narrative analysis revealed that fathers constructed surrogacy as a perilous quest that required strong intentionality to undertake. The first year of parenthood was conceptualised alternately as a joyful experience and/or one that challenged fathers’ identities and mental health. A relational framework was applied to better conceptualise the fathers’ narratives, revealing that actual connections—and the potentials for links—considerably shaped experiences of surrogacy, perinatal distress and recovery. Implications for research and policy are discussed.

## Introduction

In tandem with both the increased number and visibility of gay, lesbian, bisexual, trans, and queer or questioning (LGBTQ+) parents [1], scholars are paying increasing attention to questions around LGBTQ+ families and parenthood [2]. Although this body of research is diverse, one important strand centres around experiences of surrogacy—involving a third-party in reproduction where usually a woman carries and delivers a child for another couple or individual [3]—leading to parenthood among gay men and lived experiences of gay fatherhood [4, 5]. Indeed, scholars have noted a rise in the number of gay fathers who are building families via surrogacy [6], which can be attributed to developments in technologies of reproduction, changing legislative climates around adoption, and greater acceptance of LGBTQ+ families [7]. Yet, despite scholarly attention, much of the extant research remains constrained by applications of heteronormativity as the benchmark against which gay fatherhood is assessed and evaluated [8], rather than centring gay fatherhood itself.

This is particularly important not only because comparisons of gay fatherhood to heterosexual families reinforces heterosexuality as the preferred mode, but also because it renders invisible pathways into—and trajectories of—parenthood among gay men [8, 9]. Fortunately, an emergent body of research has sought to centre experiences of gay fatherhood via surrogacy [10]. In particular, this body of work has highlighted the range of experiences, issues, and factors that affect gay fatherhood prior to deciding to have children (i.e., procreative consciousness), during and immediately after birth (i.e., procreative responsibility), and in the months after the birth of a child (i.e., procreative transitioning). Such work, and the theoretical modelling of Fantus and Newman [10], has served to highlight the wide range of unique experiences, issues, and factors that shape gay men’s procreative identities in the context of surrogacy. To date, however, much of the extant research on gay fatherhood has tended to focus on issues of resilience, with much less work considering experiences of psychological distress [11].

### Psychological distress

Certainly, there is mounting evidence that a substantial proportion of expectant and new (presumed heterosexual) fathers experience significant psychological distress and symptoms of mental ill-health during the perinatal period [12, 13]. For instance, meta-analyses have estimated the rate of perinatal depression to be approximately 8 to 10% in new and expectant fathers [14–16]. This is concerning not only because of the substantive costs of paternal mental ill-health on national economies [17], but also because of elevated risk of harm to fathers themselves (e.g., self-harm and suicide) and their children (e.g., poorer father-infant attachment) [18–20]. Paternal psychological distress is also associated with poorer longer-term behavioural, cognitive, and physical development in children [21] and relational impacts, including increased risk of maternal psychological distress [22].

Increasingly, scholars have drawn on theories of hegemonic masculinity—a relational conceptualisation of masculinity that highlights the contribution of others to the production of gender, focusing on unequal and competitive gendered relations between women and men, and among men themselves [23, 24]—as a window into understanding experiences of paternal psychological distress. For instance, contributing factors to paternal psychological distress often include restrictions placed on acceptable masculinity, conflicting demands between work and family life, and discrepancies between the expectancies and realities of heterosexual parenthood [25–27]. Additionally, despite the wide range of experiences of paternal psychological distress [28, 29], heterosexual fathers are encouraged to either circumvent or hide their symptoms for fear of being stigmatised as weak, or alternatively valorise their masculinity in the articulation of their distress (e.g., by prioritising responsibilities towards partners and children, ahead of paternal needs) [30].

To the extent that heterosexual fathers experience and manifest psychological distress differently to mothers, practitioners working with men may struggle to distinguish male distress from traditional masculine traits, such as being stoic, expressing aggression, or taking risks [30]. Indeed, heterosexual fathers—like other men with experiences of vulnerabilities and frustrations about receiving the help they need [31]—are often reluctant to disclose symptoms of psychological distress, which in turn contributes to feelings of abandonment, helplessness, and invisibility within healthcare settings [30, 32, 33]. Such disengagement from family-centred services may also contribute to paternal detachment from the family unit, which impacts on effective parenting and reinforces the normalisation and concealment of distress in fatherhood [30]. Access to appropriate parent-facing healthcare services may also be compromised by practitioner training deficits in male presentations of distress [34], prioritisation of maternal care or a lack of non-targeted support, not to mention professional comforts in working with fathers [35]. These issues are also likely compounded by masculinised societal constructions of fatherhood, which normalise experiences of paternal psychological distress [36] and, in turn, impedes fathers seeking and obtaining the help they need [37, 38].

### Psychological distress in gay fathers

Until now, little attention has been paid to the lived reality of parenting—including experiences of psychological distress—in gay fathers [39, 40]. This omission is problematic for a number of reasons. First, from a theoretical perspective, recourse to theories of hegemonic masculinity is likely to offer new understandings of gay fathers’ experiences of parenthood and psychological distress, though research is needed to understand the ways in which considerations of masculinity shape the gay father experience [41]. Certainly, there is some evidence that heteronormativity (i.e., a hierarchy in society that privileges heterosexual ways of behaviour and frequently a central assumption of hegemonic masculinity) and attendant stigma are key sources of distress for gay fathers [42, 43], particularly for those at the intersection of constructions of race and single fatherhood [44]. Moreover, gay fathers’ encounters of homophobia—in the form of daily microaggressions, the complexities of disclosure of surrogacy, and repeatedly having to “come out” due to heteronormative biases—may also contribute to experiences of distress [10]. Thus, more needs to be done to understand the ways in which gay fathers’ experiences of surrogacy and early parenthood both require a negotiation with others when it comes to masculinity, as well as the potentials to challenge hegemony [44, 45].

Second, there is some evidence that gay fathers may experience particular difficulties negotiating the transition to fatherhood, especially in the context of heteronormativity [46]. For instance, research has shown how some men struggle to negotiate the identity “gay father”, particularly when faced with heterosexist norms and feelings of internalised shame [47]. Third, the route to gay fatherhood via surrogacy presents unique logistical, legal, and emotional challenges [10, 39]. For instance, surrogacy often involves lengthy wait times, high levels of uncertainty, and cycles of hope and disappointment if arrangements fail [48]. Additionally, surrogacy is expensive (e.g., travel and legal costs) and emotionally draining (fathers are distanced from the foetus throughout, both of which can have substantive impacts on the psychological well-being of gay fathers) [49].

Finally, the specific sociocultural context in which gay fatherhood is enacted may also pose specific challenges for gay fathers and their mental health [40]. In Israel—where the present study is situated—mainstream society is typically described as family-oriented and pro-natalist, with parenthood constructed as a route to social acceptance by honouring child-rearing practices [50]. At the same time, Israeli legislation has historically made it difficult for gay men to form families (same-sex marriage remains illegal) and become parents. Until July 2021, it was illegal for gay couples and single men to access surrogacy services [51, 52], meaning gay men conceiving via surrogacy were forced to use international services mainly in Southeast Asia or in parts of North America or Canada [11]. The Israeli Supreme Court has now ruled to overturn discriminatory laws preventing access to surrogacy services, a ruling which was met with hostility from ultra-Orthodox Jewish political parties [53]. Avenues to adoption are also limited: same-sex couples are legally prohibited from adopting a child together in Israel. Gay Israeli men—in a country with strong family values but legal barriers to same-sex parenthood—may therefore harbour both a strong desire for children and heightened awareness of the challenges of realising this wish [54, 55].

Aside from the need for sociocultural specificity, there is also a need to consider specific experiences in temporality. For instance, recent research has shown how the COVID-19 pandemic exacerbated the uncertainty of the surrogacy process, with usual practices of travelling to visit the surrogate, attend the birth, and return home with the infant, compromised by lockdowns, travel restrictions, and isolation requirements [56]. Prohibition of visits from family and friends following the birth has also been found to precipitate increased isolation, worry, and fear [57, 58]. Likewise, the ban on playgroups and childcare during lockdowns negatively impacted family well-being [59], with research showing that parents experienced elevated levels of depression, anxiety, parental burnout, and in some cases, post-traumatic stress disorder [60]. However, how the pandemic may have impacted experiences of gay fatherhood currently remains underexplored [11].

### The present study

In view of review above, we sought to better understand experiences of surrogacy, early parenthood, and mental health in Israeli gay fathers and in the context of the COVID-19 pandemic. Specifically, we took an epistemologically relational approach to ask the question: Among gay fathers, how does distress develop, organise, and transform in relation to others in the experience of surrogacy? As indicated above, hegemonic masculinity is an inherently dynamic relational theory that relies on the participation of others, even if hegemony (locally, the most esteemed way of being a man) is frequently characterised by scholars as an individualised, static, and/or toxic state in the scholarly literature [61]. Surrogacy and parenting, too, are inherently social processes, involving the creation of a child, where the potential for emotional bonds involve not just the triad of surrogate, parent(s), and anticipated child, but also others (e.g., grandparents). The “relational turn” in scholarship occurring in a wide range of disciplines involves an overcoming the old arguments of individualism versus collectivism, to instead focus our analyses on affective (i.e., states of being) relational categories as the important part of social reality [62].

Said another way, the social world can be seen to consist of mutually constituting relations rather than separated out individuals [63]. In this way of thinking, selves are “interactants”—they are transactional rather than individually agentic [64]. This way of thinking highlights human interdependency and vulnerability; whether we acknowledge it or not, our relationships can harm us. For fathers using surrogacy, this means that their affective realities are necessarily emergent and fragile, where relations may be marked by their absence or potential, as much as their actual presence. Realities are constantly assembled and experienced via transactions (or the lack thereof) involving partners, families, infants, surrogates, and others, as well as non-human entities like policies, institutions, and their homophobia. In particular, we draw on Elias’s [65] notion of valencies, which posits that “each individual has open valencies ready to connect with those of other individuals” (p. 170), the schema for which will have been shaped by early family and subsequent relations. We also draw on relational-cultural theory, which argues that humans grow through and towards connection [66, 67].

## Methods

### Overview

This interpretative qualitative study involved a secondary analysis of narratives collected through semi-structured, one-to-one interviews. The study was reviewed and approved by the Ethics Committee at The Academic College of Tel Aviv-Yaffo, Israel (approval code: 2020153) and all participants provided written informed consent.

### Sampling and participants

Participants were recruited using homogeneity purposive sampling, a non-random method where participant characteristics are defined before recruitment [68]. Limiting the pool of participants to those with similar lived experience allowed for examination of one specific phenomenon closely, whilst appreciating the individuality of each account. This is suitable for narrative analysis, which aims to capture a rich, deep picture of one particular phenomenon, acknowledging how each individual constructs their narrative of this experience [69]. Specifically, participants in the present study were Israeli gay men who had become fathers through surrogacy and who had experienced psychological distress during the transition to parenthood. Participants had to be Israeli residents and citizens and able to be interviewed in Hebrew. Fathers whose distress made it impossible for them to consent were excluded.

Participants were recruited between September 2020 and June 2021 via an advert on Internet forums, surrogacy Facebook groups, and in Meir Garden, a popular venue in Tel-Aviv among LGBTQ individuals, including gay fathers. Seventeen responses were received and those who replied were sent an email informing them about the study. Two respondents declined to participate at this point, citing time constraints. Nevertheless, a sample of 15 was considered sufficient for this study to reach data saturation [70]. Although deployed in different ways and not without controversy, saturation (i.e., generally meaning that no new information of interest to the study is being elaborated at interview) is commonly used as a criterion for determining an adequate sample size and thus the quality and credibility of a study [71, 72]. It has previously been established that saturation can be reached by around 12 participants, and certainly by 15 [73, 74]. The richness of the data we collected, with its detailed accounts, and the ways in which participants were similar (e.g., mostly secular) means that saturation is likely to occur relatively early in fieldwork [75]. Indeed, this was the case in the present study, where we found we needed to interview 15 men to achieve saturation.

Participants provided written informed consent and completed a demographic survey before taking part in one-to-one interviews. All participants were interviewed alone about their own individual experiences. Six of the participants interviewed were in relationships with other participants, specifically men with the following pseudonyms: Pasach and Avi; Abram and Malachi; and Calev and Samuel. The remaining nine participants were not in relationships with other people in the study, although most were in relationships with men not interviewed as part of the study. Participant demographics are reported in Table 1.

**Table 1:** Participant demographic information.

**Age (years)**	30–34	5 (33%)
	35–39	6 (40%)
	40–44	2 (13%)
	45–49	2 (13%)
**Education**	High school	1 (7%)
	Undergraduate	8 (53%)
	Postgraduate	5 (33%)
	Declined to say	1 (7%)
**Birthplace**	Israel	10 (66%)
	United States	3 (20%)
	Europe	1 (7%)
	Declined to say	1 (7%)
**Religious background**	Secular	13 (87%)
	Conservative	1 (7%)
	Declined to say	1 (7%)
**Age of youngest child**	0–6 months	2 (13%)
	7–12 months	3 (20%)
	13–17 months	3 (20%)
	18–24 months	3 (20%)
	25–30 months	2 (13%)
	31–36 months	2 (13%)

### Data collection

Interviews were semi-structured and narrative in style, encouraging participants to select, organise, and evaluate meaningful events to construct their experience of surrogacy and parenthood [76]. An interview schedule—developed between the authors following engagement with the literature—guided the conversations, encouraging participants to talk about their journey to parenthood and its challenges in their own way. The schedule’s flexibility allowed the interviewer to adapt the question sequence and ask for more detail. It also promoted sensitivity, reminding participants regularly that they could stop at any time. Member checking was conducted by circling back to clarify participants’ interpretations, understandings, and meanings.

Interviews lasted on average an hour, were audio-recorded, and took place on encrypted video-conferencing software, ensuring confidentiality and eliminating COVID-19 risk. The interviews were conducted in Hebrew by two trained researchers with experience in qualitative research at the second author’s institution. Participants were fully debriefed, offered a list of mental health resources, and invited to contact the researchers if questions arose after the interview. All interviews were digitally transcribed, manually translated into English by a confidentiality-bound transcription service, and pseudonymised before being received for secondary analysis by a research team not originally involved in the study. Secondary analysis is the practice of using data from already existing studies for the purpose of research [77]. Originally, the aim of the research was to investigate experiences of psychological distress among Israeli gay men who had become fathers through surrogacy. The present study used the complete dataset to investigate the specific relational-focused research question originating from the new research team; that is, understanding gay fathers’ perinatal distress by focusing on the transactional and affective nature of their emergent vulnerabilities. We involved the original research team in the analysis and writing up, as a means to bring to the work “insider knowledge” and greater sensitivity to the data [77].

### Data analysis

Johnston’s [78] evaluative steps for secondary analysis were followed, namely designing a research question appropriate to the dataset; understanding the purpose, method, and timescale of data collection, and; establishing a relationship with the original research team. The first author was in contact with all authors (i.e., the full research team) throughout to minimise the limitations of secondary analysis, which predominantly revolve around rigour and familiarity with the dataset [79]. Additionally, the narrow timeframe meant data collection and secondary analysis occurred in similar social, cultural, and political realities, aiding interpretation [80]. Finally, as per Thorne’s [81] recommendation to increase rigour in secondary analysis, the first author kept a reflective journal.

Data was interpreted using narrative analysis, which centres story as its object of investigation, prioritising an understanding of events through the way they are constructed by participants [76, 82]. While the definition of narrative is complex and plural [83], for the present purposes we defined narrative analysis as analytic interpretation of stories constructed by participants, focussing on how those stories are built, while acknowledging wider cultural frameworks within which the stories operate [76]. This approach prioritised “sequence and consequence” [83], examining how events were chosen, assembled, connected, and evaluated as meaningful. This approach allowed accounts to be understood in their fullness, rather than being “fractured” into incomplete units, as may have been the case with, for example, thematic analysis [76]. The social constructivist framework vital to the epistemological position of narrative analysis also lent itself to the secondary analysis. Narratives were viewed as co-constructed by the participant, interviewer, translator, and author. The plurality of co-creation was observed throughout, with careful attention paid to the first author’s national viewpoint and value system, which was important to note in engaging with the stories of Israeli men.

All transcripts were read equally, with all participants’ narratives informing the creation of the four overall narrative themes described below. Transcripts were read as hard copies. Initial readings were done out-loud, to *hear* the narratives, and inhabit the reflective posture of *in-dwelling* most strongly [84]. This was particularly important given the secondary nature of the analysis. Notes were made of core narratives in each interview, as well as of the first author’s reactions and emotions, to evaluate positionality. During subsequent re-reads, narrative threads were highlighted and noted in the margins. An inductive approach allowed narratives to emerge from text blocks of different lengths. Similar narrative threads were grouped and compared with the initially-noted core narratives. From this comparison, six overall narrative themes were developed, before being consolidated as four. They were reviewed as transcripts were re-read to check for any missing or hidden concepts. Throughout, the first author went back and forth between the data and the literature, and a relational conceptual framework grew from this process. This framework emerged as the most appropriate for elucidating the fathers’ accounts. All authors were involved in debating, co-constructing, and finalising the interpretation of the codes and stories. In sum, our qualitative research approach explored complexities, including the development of constructs where our interdisciplinary team worked through thorny challenges and debates to arrive at an agreement about the findings and their legitimacy [85]. This process is important to the rigour and credibility of qualitative research.

## Results

### Overview

The 15 narrative interviews followed men from their decision-making process to successfully having children. Those who struggled during the pregnancy seemed mostly to suffer from anxiety; those who struggled after reported depressive symptoms. Some spoke of less typical symptoms, such as aggression and reluctance to express emotion. All men also expressed finding happiness and fulfilment in fatherhood. Four narrative themes are elaborated: (1) intentionality and relationality; (2) a perilous quest; (3) distress and the reconstruction of identity, and; (4) pride and legacy.

### Intentionality and relationality: “Parenthood that arrives out of difficulty”

Participants expressed varying intentionality surrounding their decision to become parents. Several fathers always knew they wanted children; for others, the realisation was emergent. Pasach expressed strong conviction, saying he and his partner knew “since the beginning” that they wanted children, without considering logistics or “the genetic aspect”, and focusing only on their strong desire to “bring good people into this world”. On the other hand, Jonah “was never sure whether I would either have or not have children”, feeling through his twenties and thirties that it did not seem “like an urgent matter in [his] life”. More recently he experienced a gradual change in how he related both to his inner life and the prospect of children:

I began to feel more and more that I am in this place in life. I remember that one day… I suddenly viewed things… that seemed central and fulfilling in my life… [as] meaningless and empty… And I just began to view children differently, my life and what I desire. My thought in this matter shifted… I think it was quite related to my therapy… it started occupying an expandingly larger portion of my soul…

Only once this affective decision had been reached did Jonah reflect on the practicalities, considering financial and practical support. Logistical considerations in decision-making developed in all participants’ accounts. Narratives conveyed a sense that “gays in Israel have the longest preparation process” (Avi), as it took so long to convert the intention of having children into reality. Shlomo expanded:

We always knew we wanted children. It’s hard to decide—timing, finances, because it requires full dedication to the process, travelling a lot to the U.S., finding a surrogate, egg donation. It could take a few years.

Deciding and planning went hand in hand: participants needed to feel emotionally ready and practically prepared. They moved towards readiness in several ways: by talking to friends, other parents, psychologists, and surrogacy agencies; browsing parenting and surrogacy threads on social media; exploring shared parenting; saving money; waiting for promotions; or moving homes. Binyamin characterised this period as a trajectory:

Along the way it was a few years of learning, conventions, reading every relevant Facebook post, collecting any information that comes up in the comments, hunting down any couple in our situation or further along the track, to learn from.

The bureaucratic, logistical, and financial difficulties of having a child as a gay couple was a dominant feature in all participants’ narratives. Many made links between the difficulty in becoming parents as gay men, strong intentionality, and the anticipated relationship with the child, as Pasach highlighted:

It is parenthood that arrives out of difficulty, a challenge. I always say [my child] was chosen. He did not arrive by accident… He arrived following a mental upheaval and he is a desired, extremely loved child who we expected for a long time.

Intentionality and considerations of how to relate to children extended beyond the surrogacy period. Pasach, for example, said that “this week, actually, we are trying to develop our parenthood as working parents”. It was important for him to parent in a thoughtful, reflective manner, despite pressures of work. In this way, intentionality is a theme of gay parenting more broadly, both before and well beyond the birth.

Across all narratives, there was strength derived from intentionality. As Binyamin commented, gay fathers do not become parents by accident, or before they are ready: they are people who “planned this move for so many years… who truly experience it as amazing”. Jonah reflected on potential future challenges, acknowledging that he did not know how his child would react to the absence of a particular relationship, when they “discuss the fact that he has no mother”. His imagined answer emphasised positive intentionality:

I will not tell him that he was delivered by a stork. I will explain all about how he was created. I think it’s pretty cool to understand that people worked so hard for you to exist. That they devoted a lot of time, effort, money, understanding and intention for you to arrive.

### A perilous quest: “The path turned rocky”

Participant narratives revealed a perilous quest fathers had to undertake. Isaac explained: “You discover a whole new world… until entering, you do not realise how complicated it is”. Participants constructed this process using various metaphors: a staircase, a rocky path, a journey, or a roller-coaster. They described multiple stages, complicated by the international nature of the process, and for some, the COVID-19 pandemic. Many conceptualised distinct stages of the quest, allowing them to compartmentalise tasks involved to avoid feeling overwhelmed. Isaac explained:

If you’re currently waiting for the surrogate for 8 months, this is what you’re busy with… You don’t think about pregnancy questions—you are currently dealing with this. When you need to choose a donor, then you deal with the madness of selecting a donor, and I think there is something about it, at least for me personally, this conception made the process easier to cope with. One thing at a time.

Quests were not one forward motion, but a series of movements backwards and forwards. Some participants characterised the process not in terms of milestones achieved, but in terms of hurdles presented, demonstrating the extent to which setbacks shaped their experience. Narratives intersected with setbacks on both a personal level (e.g., surrogates withdrawing) and a global level (e.g., Trump succeeding Obama and eliminating insurance covering postnatal hospitalisation).

Some fathers knew from the outset that it would be a difficult journey. Others realised during or after. Abram used the language of a physical journey to describe his gradually developing awareness of the trauma involved in surrogacy:

[My partner] brought up the idea, and I went along, and we headed out on the way. It was exciting, I was happy to head out, but the path turned rocky, it was a nerve-wracking process. Terrible. Horrible and extremely tough that lasted 4 years.

He explained that his child was born on the thirteenth attempt, characterising this period as “years and years and years of failures and shattered hopes”. He, like others, described almost giving up: continuous loss through miscarriages made coping financially and emotionally appear almost impossible.

Participants depicted the highly relational—and thus fraught—process of surrogacy as holding no guarantee of success. They felt “anything could flash a red light” and you could only “wait” for and “receive” news (Shlomo). Several participants used the language of fortune and fate, positioning themselves as powerless to control a process involving destiny. Shlomo exemplified this, explaining, “you need the entire cosmos to converge for everything to go well”. The lack of certainty engendered anxieties, which could manifest as a desire to control everything possible. Participants constructed the journey as a series of choices (e.g., choosing a surrogate and egg donor), where getting it wrong risked everything. Underneath these attempts at control, however, participants understood they had little power to ensure a healthy baby would arrive. This induced fear in many narratives, which was strongest for those who could not raise more money. Shlomo explained:

There are fears and concerns that you will lack finances and need more money. Insurances do not cover miscarriages, God forbid, nor premature intensive care units, and that is a fear that accompanies the entire journey up until the day of delivery… There are no second chances here, to be precise. I’m not undermining the process of straight people… but the recovery process, even if it’s a year or two… after that you can just try to conceive again, there is no financial aspect you need to recover from.

Fathers also found the physical distance—and thus absence of relational cues to closeness—between themselves and their foetus particularly difficult, producing a sense of separation and longing to be nearer. Some tried to bridge the gap by consuming media about pregnancy (e.g., fertility podcasts, Instagram stories of pregnant influencers). The fact that the pregnancy was happening in a different country, time zone and language, also created a sense of remoteness and associated anxiety. Shlomo explained that when his surrogate had a uterine cyst, he did not know what it meant which, “affect[ed] us and cause[d] us distress”. Whereas, in a biological pregnancy, ailments could be felt, assessed for pain, and often treated—with the help of healthcare practitioners—by the pregnant person, the fathers were relatively powerless. To alleviate fear, participants relied on pregnant/postpartum women around them to contextualise medical issues. Communication and connections with surrogates also emerged as crucial in easing anxiety, as Binyamin’s words demonstrate:

There could definitely have been more moments [of distress] had we gone through the process with someone else, if [our surrogate] hadn’t shared and been so into it, sharing things of her own initiative. Because sometimes we didn’t know what to ask, since it was our first experience. So, she would share what she experienced and how she felt, and the tests that needed to be done.

Relationships with surrogates made clear that closing the distance between fathers and surrogate—with trust, information, and communication—could be a tonic.

The COVID-19 pandemic compounded the quest’s difficulty, complicating the logistics of attending the birth. Isaac’s narrative—which contrasted his experiences of having a baby before the pandemic and during the pandemic—illuminated this. Being abroad with his first child was “a period of grace”; with his second it was “180 degrees different”. Participants experienced severe anxiety that they would not be able to travel. Isaac found that even when travelling became possible, the anxiety he was feeling ensured there was something else to fret over:

It was an experience of anxiety, great distress, and uncertainty, that lasted approximately 3 months until flying. From the moment we boarded the flight, it was a different type of anxiety. Because you say: ‘Okay, I am on an airplane, so I will be there during the delivery’. But you are scared because you are in a foreign place, it is not like the Israeli health system. I do not know anyone, what happens if I get infected? So, it is very stressful and also very lonely, it was only us…

After babies arrived, the pandemic continued to impact participants. They had to isolate upon arrival to the United States and Israel, could only go out for walks, and could not see anyone face-to-face. Some were not allowed in the delivery room for the birth. Isaac viewed these stressors as cumulative:

I will try to think if there was a certain moment. Meaning, the thing that frustrates so much during this period, is the fact that it does not end… meaning, it just keeps accompanying and you do not really see the end of it… the COVID-19 pandemic.

When the quest finally ended with the arrival of healthy babies, participants all felt they had achieved something incredible, making the hardship worthwhile. Shlomo summed up this feeling, expressing his pride at having “contain[ed] and cope[d] with” something “insane”.

### Distress and the reconstruction of identity: “Pushed to the edge”

All participants spoke about the routine ways a new baby impacts life: sleep deprivation, lack of free time, loss of time for previous hobbies, and lack of time for the couple’s romantic and sexual relationship. Isaac constructed the change as both practical (“a collection of endless things to do”), as well as having a “more essential and deeper aspect”. He described the affective shift involved in realising his new obligations to another:

Parenting has something… a sort of load on your shoulders. And now you sink with this burden because it is a very, very great responsibility. It is something that is hard to conceptualise, but it is present in every layer of your life. The responsibility, the worry, add another sort of dimension, between depth and heaviness of something that I now have, that I am responsible for. This thing is very serious. So, it really, really changes your life.

Several participants constructed the change as fundamental and even biological. For example, Samuel used a documentary about parental brain neural alteration to explain how the change felt for him.

For certain fathers, the emergent self involved in fatherhood constituted a period of emotional upheaval. This was sometimes amplified by the travel involved in surrogacy. Avi, for example, found it difficult to adjust to the label of “father” in two different countries. In the United States, spending time alone with his partner and child, he experienced a “sense of perfect harmony” and believed “that it was very natural to me, that I was born to be a father”. Returning to Israel—where he had grown up believing gay men could only have children via a closeted heterosexual life—ruptured his fatherhood identity. He constructed returning home as rebuilding “everything from scratch”, which led to him feeling that “everything was falling apart”:

Personally, when we came back home, the house felt estranged because I initially raised [my son] for the first month of his life in a foreign country, a different apartment. But this was arriving at a house I know, but in a different role, with another person, and it turned into a very foreign place. I felt as though… I don’t know how to describe it, that I was in an estranged alien place I must readjust to, become accustomed to my title as a father, and in retrospect, I realised that I was suffering from postpartum depression.

Other men’s experiences of psychological distress centred on their relationship with their babies. Fathers talked about difficulties bonding and not feeling “as if we were flooded with joy and love for the kids from day one” (Shlomo). One link to distress that emerged was the day-to-day difficulty of parenthood, which participants saw as a potential barrier to feeling close to their children. This pattern played out in Abram’s narrative: he became “traumatised” from his new-born’s constant crying. He described feeling distressed “all the time”; even on the nights when it was not his turn to wake up for the baby, as he panicked about the next night. Abram’s narrative painted a picture of hopelessness, driven by his fear of the baby crying. He felt “incapable of recruiting my soul’s positive forces”, and reflected that even at the time of interview it “is still hard for me to hear him cry”.

Certain depressive symptoms reported by participants were typical of postnatal distress regardless of gender and sexual orientation, such as not being able to get out of bed, hopelessness, and feeling disconnected from loved ones. Others were more male-typical symptoms, such as feeling numb or experiencing rage. Several men remembered moments where they felt like throwing their child as a symptom of extreme frustration and being “pushed to the edge” (Samuel). Samuel remembered a parenting workshop leader explaining how to deal with rage: “if the baby cries a lot… do not shake the baby, but lay them down and leave the room for a couple of minutes to take a deep breath”. At the time, Samuel was scornful of how obvious this advice was, but in retrospect acknowledged it was important.

Other men explained how they suppressed their feelings to cope, throwing themselves into jobs and household tasks instead. Uri considered his “practical and mission-driven” approach as unhealthy in retrospect, noticing that suppressing feelings led to his distress emerging in ways he could not control. He spoke about “disproportionate reactions” to everyday frustrations, which alerted him to the fact that, underneath his calm exterior, he felt “grief about the life that [he] had and lost”.

Only two participants—Avi and Abram—labelled their experience explicitly as postnatal depression. Both their narratives illuminated poor understanding of the condition in their social worlds. Abram’s phrasing that he “truly felt as if [he] had postpartum depression”, revealed some uncertainty in staking a claim to the condition. Similarly, when Avi tried to discuss the condition with his friends, he “could not name it”, knowing only it was “something unfamiliar”. This uncertainty added to the feeling of isolation:

People were not so aware of what I experienced. I did not understand what was going on with me… [My partner] …did not comprehend what was going on with me, and neither did I. So, we experienced miscommunication, and it was not pleasant. I felt that no one understands what was going on with me. (Avi)

Partners of men suffering with postnatal depression could struggle to recognise what was happening. They described frustration and an inability to communicate with or help their partners. Recognition of postnatal depression emerged as key to being able to support their partner and cope themselves, as Malachi—Abram’s partner—noted:

I just realised that Abram was experiencing a mental difficulty beyond the existing physical one, and that first—I need to be non-judgmental towards him, because this is not something he chose, and it is out of his control. I just need to comprehend, contain him, step in his shoes, with utmost possible empathy, and see how I may help.

Various avenues were reported as important for recovery from postnatal depression: therapy, self-care, hobbies, sourcing childcare so participants could go out, and communication and connection with partners, friends, and family. Additionally, fathers found positive time spent with their baby helpful. Abram’s account of his journey towards recovery elucidates the connection that formed alongside postpartum depression easing.

Some people love their child immediately after he is born. For some people, it takes a bit more time, and sometimes it might take a while. There is no need for self-flagellation, because you know, it was strange for me, I did not have any exceptional love when I first saw him, or fireworks. The opposite—I felt irritated, helpless… and time goes by, day by day… that connection formed.

All participants’ narratives, regardless of the level of distress they experienced, emphasised the importance of weaving a thread from the old self to the new self as a father. Many did this by creating time for activities that fulfilled them before they had children, including exercise, yoga, meditation, music, and eating out. Overall, participants felt their lives changed completely with the birth of their child, but that as time went on, their routine settled and they could connect to previous activities and identities, which supported well-being.

### Pride and legacy: “They now think differently”

All participants constructed narratives of accomplishment and pride, typified by a conversation Pasach remembered with an employer’s child:

My former boss… introduced me to her son and said: ‘This is Pasach, he has a husband and child by surrogacy. Do you know what surrogacy is?’ He is a child in fourth grade! I had the chills. And the kid said: ‘O.K.’ and moved on. It did not constitute any drama or anything. In a larger circle, there could be a gay child in his class, someone who freely came out of the closet, someone childfree, someone in a relationship. When I grew up, a gay person was compared to someone who will die alone.

Pasach’s embodied affective reaction mirrors his emotional response to the mother’s frank, nonchalant explanation, and the child’s easy acceptance of his situation. His assertion that future queer people will be able to come out “freely” illustrates his pride in challenging homophobia and contributing to normalising gay-parent families.

Overall, the fathers received positive reactions to their family formation, rarely encountering homophobia, despite anticipating it, as Isaac summarised:

The attitude we have been experiencing is amazing… I am even positively surprised. We receive a lot of support everywhere we arrive. There is great support and great empathy. I never encountered negative reactions anywhere. There is something about them being children, that neutralises homophobic people. Meaning, once children are involved, everyone becomes very positive.

Almost all participants told stories of family members coming to the airport or their home, excited to meet the child(ren). In situations of tension, the men more often had stories of movement towards resolution than they did of lasting rifts. However, participants still encountered microaggressions and found bureaucratic systems exclusionary. A common microaggression was unwanted parenting advice, with participants divided on how frustrating they found this, from highly offensive to a minor annoyance. Levi reported developing an ability to “screen” interfering comments, to stay “focused on…what you think is right”. Avi described being asked often who the “biological parent” is as “very difficult” and “so inappropriate”. He responded by making people feel uncomfortable, to alert them to the disrespect their question implied by construing parental legitimacy through heterosexual biology. Several fathers encountered the belief that a child needs a mother from gay friends (suggesting internalised homophobia to participants) or parents, painting a picture of broadly supportive families who made occasional unhelpful, heteronormative comments. Participants also told stories of their family formation changing their parents’ minds: Samuel’s parents “now think differently”, seeing that a child can be raised excellently by two men.

When it came to bureaucracy, the picture was also nuanced. Some had a positive experience, such as Uri, who was aided by his background as a lawyer:

The Ministry of Interior—the person was so politically correct while speaking with us, to the point that I was wondering whether he went through training. He wanted to ask who [our son] belongs to, but then apologised and amended: ‘I did not mean that. I meant that he belongs to both of you, but I referred to the genetic aspect…’

More commonly, fathers found the bureaucratic process difficult. The extreme length and “draconian” rules “generated… rejection feelings [and] unbelonging” for Samuel. Shlomo expanded:

There is no justification that upon my return, only one child is registered as mine, and the other must be adopted by me. This is unfounded, it is completely unreasonable to have only one child recognised in the official documentation, and the other child misrecognised by the Ministry of Interior, you cannot report this child to the tax authorities, it makes no sense.

The narratives constructed systems as clunky and counter-intuitive for gay fathers, as they were built for heterosexual, biological conception. Some participants noticed the same pattern at work:

Parental leave: I informed someone at human resources about the pregnancy and told her, ‘Give me a hug’ or something, and the first thing she said to me was, ‘I do not think you are eligible for parental leave…’ Although they pay full regular salaries to women on parental leave, in my situation she said no. (Noam)

Participants indicated a range of support they would like to see to make gay parenthood easier. Financial support was a priority, as currently only the most affluent gay couples could have children. Channels of information-sharing were suggested: psychological support groups; online workshops; and a specific guidebook for the gay community on surrogacy and parenting.

Participants felt services should predominantly be for all family types, with several participants wanting to be treated exactly the same as heterosexual-parent families. Others found services designed specifically for gay-parent families offered more tailored advice and a sense of belonging. Noam found gay parenting workshops helpful because of their specificity, as outlined in the first quote below. The value in creating safe spaces for gay parents to discuss things uniquely affecting their family is discussed by Uri in the second quote about an online environment:

[It covered] specific topics of men’s parenting. How to define the nuclear family with the child, what are the boundaries. Whether my mother or sister have the authority to tell [my child] things… Because she is a mother or woman?

It is an extremely supportive community, I must say. Starting with surrogacy, and then parenthood. There are Facebook groups, and everyone is very happy to help, want to, and every answer receives 20 replies, private messages, etc.

The need for safe environments can be gleaned in the participants’ stories of negative experiences. Isaac attended parent-and-baby events and was asked if he was sent by his wife. It made him feel “very, very much that I do not belong in the situation… I felt a bit lonely there”. It left him with a desire for a group with parents “just like me”, in order to feel “comfortable”. Similarly, Samuel’s partner tried to join a mothers’ Facebook group, but was not accepted.

Finally, several participants mentioned they had set up routes to support themselves. Many spoke about the importance of passing on information, through speaking with friends, posting on Facebook groups, meeting strangers on Zoom to discuss the surrogacy process, or setting up physical spaces for mutual support (e.g., one participant started an infant massage group for gay parents). Where the state could not cater for their needs, these fathers were proactive in creating their own support networks.

## Discussion

This study explored Israeli gay men’s transition to fatherhood, focusing on difficulties during the surrogacy process and first years of parenthood. In language that aligns with valued forms of masculinity, fathers embarked upon a quest for a child, requiring strong intentionality, that had the real potential for men to prevail and leave a legacy. For some it was difficult but not unmanageable, where others recounted it as a period that negatively affected their psychological well-being and sense of self. Once babies were born, some parents felt immediate reward and happiness, while others struggled mentally. Ultimately, all fathers achieved fulfilment and a sense of pride, contributing to a legacy of gay fatherhood beyond their own family unit.

Throughout, fathers’ relationships (or lack thereof)—with their babies, partners, surrogate, family, bureaucracy and information—could work to assuage or intensify their affective dramas and difficulties. One participant’s conception of many “people” bringing his child into existence captures this journey as the inherently relational experience it is. Elias [65] viewed the self as an ongoing process, not a stable entity: people are constantly made and remade through their interactions with others and the wider social world. People have porous boundaries rendering them capable of attaching to others, with these bonds becoming parts of self. Elias [65] proposed the concept of *valency*, whereby people are at the centre of a kind of asterisk of connections. Some stems are firmly connected to people, while others are more loosely attached; and yet others are vacant, available and seeking to bond. The fathers in this study can be viewed as having a particularly dynamic asterisk of connections, in particular between what Beier [86] deemed the core “surrogacy triad” (p. 634)—the foetus/child, surrogate, and parents—who all depend on each other in different ways for their wellbeing across the quest.

The initial absent bond between father and child, and the potential for future connection and disconnection, was a focus of all narratives. The emergent bond variably induced hope and fear, closeness and alienation, and comfort and longing. Fathers who had not experienced the trauma of failed surrogacy attempts described more comfort in allowing this bond to form during pregnancy, whilst still lamenting the physical distance involved. This echoes Ziv and Freund-Eschar’s [87] proposition that the vicarious nature of surrogate pregnancies, where parents have limited tactile contact, presents a paradox of bonding: it can be more difficult for fathers to feel like parents, whilst encouraging stronger imaginings about their future child. Fathers whose surrogate miscarried experienced any bonded valency with the foetus torn away, leaving them wounded and lacking a part of themselves. This echoes theorists who construct miscarriage as loss of a future child [88], as well as research highlighting that men experience a grief process following a miscarriage [89]. These fathers’ coping strategies during future pregnancies—distancing themselves mentally from foetuses, bracing for the worst and trying to prevent affective valencies forming—are reflected in research that addresses women with a history of miscarriage, and how they control their emotions in subsequent pregnancies [88].

Connection with the child was also crucial to recovery from distress, both from prenatal anxiety and postnatal depression. With prenatal anxiety, feeling able to prepare for the baby’s arrival (through conversations and practical planning) combatted the lack of connection and control that distance produced. For postnatal depression, finding ways to overcome isolation and relate to the baby was often cited as key to mending. Growing research positions recovery as a social process [90], whereby social interactions allow people to experience themselves as having skills as well as difficulties. Learning what their babies needed and how to placate them—signs of a strengthening valency—fostered feelings of competence in the fathers, enhancing a positive view of self, and aided recovery for those with postpartum depression. This echoes research showing talking therapy—helping people explore ways to connect with children without pressure—can be as effective as pharmacological treatment for postnatal depression [91]. It also emphasises the need to publicise more widely that father-infant bonds typically take time to form after birth [30], and more so if they are disrupted by depression [92].

The men constructed their connection to their child as one where genetics held varying levels of importance. For Nebeling Petersen [48], this is a narrative attached to other stories. On the one hand, parents’ construction of the genetic link as foundational emphasises connectivity to ancestors, both living and more imagined. The increasing closeness that some men found with their own parents after the birth of their child, contained stories of excitement at the prospect of genetic lineage. On the other hand, fathers who constructed their connection with their child as more metaphysical than genetic believed non-genetic fathers claim to parenthood should be validated, and expanded on the narrative in the gay community of queering family connections [48]. Questions from outsiders about genetics—which regularly offended participants—were interpreted as challenging the validity of men’s relationship with the child. Fathers’ rebuttals to these questions contributed to queering parenthood [93].

Throughout the narratives, partners were constructed as primary sources of support, and in masculine terms, teammates on the quest, and in relational terms, integral parts of self. This was important in responding to the pressures of surrogacy, especially where the difficulties of postnatal depression presented [30]. Here, partners who legitimise the reality of postnatal depression (e.g., father not to blame, a temporary distressing and disconnected state) were helpful. The social isolation of lockdown may have amplified the cut-off from relations that is part of depression for some men. What constituted the outside world shrunk dramatically for participants in lockdown. Studies have consistently found that questioning the legitimacy of one’s feelings, and being under-informed about the nature of postnatal depression, are both barriers to seeking help [25, 29, 36]. This emphasises the importance of publicising postnatal depression prevalence in men, which may also aid partner recognition [13, 36].

The men’s bond with their surrogate—the final member of the ‘surrogacy triad’ [94]—ranged in strength. Participants who had frequent communication with their surrogate found this bond to be a source of comfort. Gunnarsson Payne et al.’s [95] relational review of surrogacy studies found that cultural narratives often define this relationship, which can impede connections. They suggested these narratives unhelpfully construct surrogates either as a quasi-mother who secretly wants to keep the child, or solely as a vessel. They instead suggested surrogates and parents can form an idiosyncratic unit, in which everyone’s role is well-defined but symbiotic. Shah et al. [94] suggested that this should become the norm, with research showing that when this bond is allowed to develop, both parties can demonstratively care for each other and report better experiences of surrogacy, as was the case in the present study. Experiences of surrogates in our study existed at the edges of men’s narratives. Several participants alluded to the unequal power and financial dynamics involved in surrogacy. In such a delicate relational process, where the ethical and moral considerations are complex, it is important to find a balance where queer families are given opportunities to create families, and surrogates (often less affluent than the couple; and usually women) are empowered to manage the process happening within their own bodies in ways that work for them. In positive experiences for surrogates, communication and care during the pregnancy allowed the relationship to continue beyond the infant’s birth (when desired), potentially also empowering the children involved in their understanding of their origin story [96].

Looking beyond the surrogacy triad, participants also existed within a social web of family, gay community and institutions, related to the Israeli Jewish national habitus [97], which is an embodied amalgam of worldviews, historical processes, and sensations. These have all moulded contemporary perceptions and actions in Israel [98]. Israeli society is a society of immigrants, which is highly concerned with group affiliation, questions of identity [99], and cultural traumas of attack, exile, and persecution (the most extreme example being the Holocaust). These traumas constitute the Israeli habitus around annihilation anxieties, on the one hand, and national pride on the other [100]. In response to these traumas and in order to establish a new identity, certain sociocultural myths and narratives were developed. Of these, the most important were the value of the family and image of the “Sabra”, the new Jewish population born in Israel [101]. The Kibbutz is one prominent example of communal living and child rearing in Israel. Originally formed on utopian ideas, this society had defined itself by full responsibility for the child’s wellbeing focusing on discarding the old-fashioned diaspora influenced Jew, in favor of rearing a new Sabra type [102]. It may be argued thus that adhering and adapting to the family system that is so central to the Israeli national habitus imbues gay fathers with a sense of potency, invincibility, pride [103], and a common identity with the larger society they wish to belong to [104, 105].

From a complementary angle, the existential rejection anxieties that are prevalent in Israeli society may help to underpin homophobia that is perpetuated through systems. In particular, situations of risk and identity threat (both internally or externally, such as during the pandemic), social and psychological processes of scapegoating, that is, the exclusion and rejection of marginalised groups [101], which seemingly jeopardise the heteronormative societal constructions of the family, serve as a means to restore cohesion, identity, and belonging to Israeli societal systems. This scapegoating position [101] can be understood as a special group phenomenon of interpersonal relations between the LGBTQ+ subgroup (scapegoats) and the heteronormative Israeli national habitus (scapegoaters). The scapegoats, afraid of being rejected, do their best (e.g., become fathers) to fit in and belong to the Israeli familial system, while the scapegoaters, due to centuries of persecutions and marginalisation, identify both with the fear of rejection and with the aggressors [102], and so develop (partly) unconscious dynamics of detesting and aggression towards the scapegoat’s subgroup. This cyclical process includes not only detestation towards the weak and marginal, but also an unconscious relationship with both the scapegoats’ fear of annihilation and need to belong to the scapegoater’s group. Fantus and Newman [10] observe that the cumulative impact of these instances can lead to considerable distress, by conferring a sense of marginalisation. While participants broadly viewed microaggressions in family and community settings as unpleasant but manageable—attributing them to lack of education and exposure to same-sex parent families—system-wide homophobia caused greater distress. Systems’ ability to commit microaggressions is well-documented with regards to race [106] and, increasingly, research shows that a culture’s heteronormative value system—often expressed in interactions with the state—contributes to negative mental health outcomes for LGBTQ+ individuals [107]. The fathers in this study reported heteronormative bureaucracy that added to their psychological distress, whereas systems that felt inclusive positively impacted them. Taken together, it may be argued that Israeli gay men’s experience of the transition to fatherhood illustrates the coexistence of both inclusion and rejection processes in the Israeli Jewish national habitus.

### Limitations and conclusion

Despite this study’s important focus on an under-researched population, a number of limitations should be considered. First, all participants were on high salaries, with all but two completing further education. Many could afford weekly therapy and regular childcare. Given that poverty and poor mental health are risk factors for postnatal depression and that there is an established link between poverty and poor mental health [91], it is recommended that future studies focus on gay fathers in lower socioeconomic demographics, who cannot afford regular psychological help or extensive childcare (e.g., in countries where non-commercial surrogacy is accessible to gay men and therefore more affordable). Secondly, all fathers in this study ultimately had healthy babies, which they reported made the process feel worthwhile. Men who underwent the surrogacy process unsuccessfully were not included in this study. We must therefore be wary of triumphant narrative bias in any conclusions we draw about the effect of surrogacy on men’s mental health. It is possible that men who decided to stop surrogacy attempts due to miscarriage, lack of funds, emotional burnout or other reasons, may report different experiences, and they should be included in future literature.

Thirdly, related to Israel’s military service requirement, not to mention the financial requirements of parenthood, it is likely that many Israelis start their families at a later age as compared to other young adults in other countries. This is manifested in the relatively older ages (35–49 years) of the fathers in our sample. Fourthly, all participants were Jewish and the vast majority defined themselves as secular. The similarity of the men could have shaped similiar beliefs, experiences, reactions from family, friends, and/or community, thus limiting generalisability of the findings to non-Jewish or secular fathers. Nevertheless, it is the development of concepts, specifically relationality in this study, that means qualitative research can be used to elucidate and make clear what “the data mean at a broader level, thus allowing *it* to speak across realms, sites, and contexts” (p. 1768, italics in original) [108]. Fifthly, although some men referenced the length of their relationship indirectly by talking about how its strength and depth got them through difficult times, we did not specifically measure the length of couples’ relationships, the span of which may have influenced the experiences which participants discussed. Additionally, amongst the participants there were three couples, and so it could be argued that the narratives deriving from the two interviews of the same couple are linked, as they refer to the same relationship and the same surrogacy process. However, we noted both contrasts and connections in perspectives and described experiences by within each couple. However, a dyadic qualitative analysis is beyond the scope of the current paper, yet a possible fruitful avenue of future research.

Finally, there were relatively few very “dark” instances recorded in these contemporary narratives. However, difficulties we uncovered were profound and included depression, “unequal power and financial dynamics involved in surrogacy”, and encounters with stigma, homophobia and some resistances to gay parenting. While we might be accused of “soft-pedaling” the difficulties of gay surrogacy, we were at pains to avoid overplaying the idea that gay fathers will necessarily encounter trying levels of homophobia, stigma, and so on. In line with contemporary qualitative research showing a collapse in the power of homophobia to shape men’s lives [109], the lived experience of the participants did not align to the ‘darker’ historical times encountered by gay men. As the authors included gay men on the team, we wanted to ensure the current stories had freedom to exist and breath as narratives of contemporary gay life and parenting in the 2020s, without forcing them to fit a pre-set story arc, or indeed pathological narrative.

These limitations notwithstanding, the present study provides important insights into the lived experiences of gay fathers and their experiences of surrogacy and parenthood. Their voices have rarely before contributed to the discourse on perinatal mental health, making this study particularly important. The narrative themes identified highlight how the surrogacy process produces anxiety and how the transition to parenthood produces both happiness and mental anguish. Surrogacy was easier with increased connection between the surrogacy triad. Similarly, parenthood struggles were alleviated by connection with partners, babies, families, and one’s former sense of self, as well as by regular psychological support and childcare. Paternal postnatal depression emerged as a label participants struggled to claim, though the eventual ability to claim this label contributed to recovery for some fathers. In particular, findings emphasised the inadequacy of bureaucratic and institutional systems to assist and include same-sex families, and the subsequent harmfulness to gay parents needs to be considered. The accounts described in this study indicate the need for widespread adoption of inclusive language across institutions, increased support throughout the surrogacy process, increased publicity of perinatal distress in men, and improvements to the diagnostic process of perinatal distress in men [92].
